# Accurate identification of centromere locations in yeast genomes using Hi-C

**DOI:** 10.1093/nar/gkv424

**Published:** 2015-05-04

**Authors:** Nelle Varoquaux, Ivan Liachko, Ferhat Ay, Joshua N. Burton, Jay Shendure, Maitreya J. Dunham, Jean-Philippe Vert, William S. Noble

**Affiliations:** 1 Mines ParisTech, PSL-Research University, CBIO-Centre for Computational Biology, 35 rue St Honoré 77300 Fontainebleau, France; 2 Institut Curie, Paris, F-75248, France; 3 U900, INSERM, Paris, F-75248, France; 4 Department of Genome Sciences, University of Washington, 3720 15th Ave NE, Seattle, WA 98195, USA; 5 Department of Computer Science and Engineering, University of Washington, 185 Stevens Way, Seattle, WA 98195, USA

## Abstract

Centromeres are essential for proper chromosome segregation. Despite extensive research, centromere locations in yeast genomes remain difficult to infer, and in most species they are still unknown. Recently, the chromatin conformation capture assay, Hi-C, has been re-purposed for diverse applications, including *de novo* genome assembly, deconvolution of metagenomic samples and inference of centromere locations. We describe a method, Centurion, that jointly infers the locations of all centromeres in a single genome from Hi-C data by exploiting the centromeres’ tendency to cluster in three-dimensional space. We first demonstrate the accuracy of Centurion in identifying known centromere locations from high coverage Hi-C data of budding yeast and a human malaria parasite. We then use Centurion to infer centromere locations in 14 yeast species. Across all microbes that we consider, Centurion predicts 89% of centromeres within 5 kb of their known locations. We also demonstrate the robustness of the approach in datasets with low sequencing depth. Finally, we predict centromere coordinates for six yeast species that currently lack centromere annotations. These results show that Centurion can be used for centromere identification for diverse species of yeast and possibly other microorganisms.

## INTRODUCTION

Centromeres are chromosomal regions whose function enables faithful chromosome segregation via formation of the kinetochore (1). These elements are also key regulators of genome stability (2) and replication timing (3,4). In animal and plant genomes, centromeres are large heterochromatic zones, but many yeast species have *point centromeres*, which are sequence elements as small as 125 bp (5). The relative simplicity of yeast centromeres has allowed their functional dissection, and the abundance of sequenced yeast species has shed light on the evolution of centromeric elements across hundreds of millions of years of evolution (6).

The Hemiascomycete yeasts comprise a highly important taxon of model organisms in genetics and genomics (7,8), and some are crucial in biotechnology applications such as recombinant protein expression (9). Most yeast plasmid expression systems are dependent on locating and identifying yeast centromeres because they confer the property of stable segregation to episomal plasmids (10). However, efforts to annotate yeast centromeres are hindered by the extraordinary diversity among species (11). Mapping centromeres in diverse species has been attempted, usually through phylogenetic tools (6,12) or chromatin immunoprecipitation (13). However, both approaches have drawbacks, the former due to the divergence of underlying functional motifs and the latter due to non-specific signal. A method of mapping centromeres that does not rely on evolutionary predictions or rare protein–DNA interactions would therefore be useful for identifying centromeres in novel species. These new centromere sequences could then be used, for example, to build new plasmid-based strain engineering tools in species important for research and biotechnology.

Chromosome conformation capture tools such as Hi-C and related protocols use proximity ligation and massively parallel sequencing to probe the three-dimensional (3D) architecture of chromosomes within the genome (14–16). Hi-C and related techniques create a *contact map*, consisting of a matrix of genome-wide interaction counts between pairs of loci. Contact maps have recently been shown to contain long-range contiguity information: Hi-C has been used in the scaffolding of *de novo* genome assemblies (17,18), molecular haplotyping (19) and metagenomic deconvolution (20,21). These methods have also paved the way for a more systematic analysis of genome architecture, including long-range gene regulation and chromatin architecture (22–24). These advances raise the possibility that contact maps might be used to determine the location of subchromosomal genomic structures such as centromeres and nucleoli.

A recent study attempted to map centromere locations using Hi-C contact probability maps (25). This approach exploits the strong architectural features of yeast genomes to determine centromere positions and rDNA clusters in *Saccharomyces cerevisiae*, *Naumovozyma castellii*, *Nuraishia capsulata* and *Debaryomyces hansenii*. In yeasts, centromeres are tethered by microtubules to the spindle pole body, leading to centromere clustering (24). Similar clustering is also present in other organisms, such as the parasite *Plasmodium falciparum* and the plant *Arabidopsis thaliana* (26,27). The clustering of elements creates a distinct peak of interactions between chromosomes in the *trans* Hi-C matrix, and an X-shape in the *cis*-elements of the interchromosomal contact counts Pearson correlation matrix. Marie-Nelly *et al*. (25) exploit this X-shape structure in *trans* contact counts correlation matrices to first detect a 40 kb window containing each centromere.

In a subsequent step, they carve out 40 kb-by-40 kb windows of contact counts for each pair of centromeres and refine the prediction by fitting a Gaussian on the sum of *trans* elements of these windows, a procedure similar to those used for single molecule localization or high-resolution microscopy (28). However, this method has several limitations. First, the procedure relies on the correct pre-localization of candidate centromeres. This step fails when other sequences also colocalize (for instance, rDNA sequences). Second, the last step of the procedure collapses the data of several *trans* interaction windows into a 1D profile and calls the different centromeres independently from each other, thus potentially losing some valuable information.

Here we propose a novel method, Centurion, that jointly calls all centromeres in a genome-wide Hi-C contact map. The key idea is that a joint optimization can effectively exploit the clustering of centromeres in 3D. We first compare our method to the one described by Marie-Nelly *et al*. on four publicly available high-resolution Hi-C contact maps (*S. cerevisiae* (29) and three stages of *P. falciparum* (26)). This comparison demonstrates that Centurion infers centromere positions more accurately than the previously published method. We then apply our method to Hi-C data from 14 diverse yeast species (20), yielding high-resolution centromere location predictions for each chromosome in each species. For the eight species that already have centromere annotations available, our predictions match very closely with the existing calls. For species with as-yet uncharacterized centromeres, our predictions will serve as the basis for targeted experimental validation and could be used to create new plasmid tools in these yeasts. Our results suggest that Centurion has great potential to identify the centromere locations of many yeasts for which standard techniques have failed to date. Furthermore, we demonstrate that Centurion works well even with very limited sequencing depth Hi-C libraries generated from pooled samples, making it a practical as well as powerful tool to use on single microorganisms and metagenomic mixtures. Centurion is freely available as open source software at http://cbio.ensmp.fr/centurion.

## MATERIALS AND METHODS

### Single organism Hi-C data

We use Hi-C data gathered in two previous studies: an asynchronous budding yeast (*S. cerevisiae*) sample (16) and three different stages of the human malaria parasite *P. falciparum* (26). For the budding yeast Hi-C data, we download and use the files HindIII + MspI (intra and inter) from http://noble.gs.washington.edu/proj/yeast-architecture/sup.html. For the three stages of *P. falciparum*, we download and use the Hi-C raw contact counts at 10 kb resolution from GEO archive (Accession codes: GSM1215592, GSM1215593, GSM1215594).

### Metagenomic Hi-C data

For Hi-C data from metagenomic samples we use the two synthetic mixtures (M-Y, M-3D) generated in (20). We also perform additional sequencing of the M-3D sample using two restriction enzymes that cut more frequently than the 6-bp cutters HindIII and NcoI used in the original publication. We perform these additional Hi-C experiments exactly as described in (20) with the exception that we use Sau3AI (a 4-bp cutter that recognizes ‘GATC’) and AflIII (a 6-bp cutter that recognizes ‘ACRYGT’) to fragment the DNA. We then combine the reads from these two libraries (Sau3AI and AflIII) to produce Hi-C contact maps.

We process the Hi-C libraries from these metagenomic samples in a similar fashion to the Hi-C data from the above mentioned single organism samples, with the exception of two differences. First, we map the reads to a meta-reference genome that concatenates the reference genomes of all the organisms in the corresponding sample. This mapping strategy discards contacts which cannot be uniquely assigned to a single organism, thereby reducing contamination between contact maps. Second, because of the longer read lengths for the metagenomic libraries compared to single organisms (80–101 bp versus 20–50 bp), we post-process the non-mapped reads that contain a cleavage site for the restriction enzyme used for the library generation, as previously described (30). This post-processing increases the number of informative contacts extracted from the metagenomic Hi-C libraries by 5–15% depending on the read length and the cleavage site frequency. The resulting set of informative contacts are processed further at appropriate resolution, as described below.

### Assembling the *K. wickerhamii* genome

Two input genome assemblies are used for creating the new *K. wickerhamii* reference genome. The first is the publicly available *K. wickerhamii* reference genome originally sequenced by Baker *et al*. (31), and the second is the *K. wickerhamii* associated cluster from Burton *et al*. (20). These assemblies are merged with CISA (32) and then merged using the mate-pair library from (20) using the ‘scaffold’ command from IDBA (33). Hi-C reads are then aligned to this assembly, and the seven scaffolds containing the seven *K. wickerhamii* centromeres are identified. Lastly, this assembly is run through Lachesis (17), with a restriction that the seven centromere-containing scaffolds could not be merged.

### Data normalization

Hi-C contact counts are subject to many biases (GC-content, mappability, etc.) (34). To correct for technical biases, we apply to the raw contact counts an iterative correction and eigenvector decomposition method proposed by Imakaev *et al*. (35), based on the assumption that all loci should interact equally. We then rescale the resulting matrix such that the average normalized contact count is equal to the average raw contact counts.

### Centromere calling

We segment the full genome into *N* windows of similar length (*N* = 611 for *S. cerevisiae* at 20 kb) and summarize the Hi-C data by the contact count matrix $C\in \mathbb {R}^{N \times N}$, where *C*_*ij*_ is the normalized number of physical interactions captured between loci in windows *i* and *j*. For each window *i* ∈ [1, *N*] we denote by *B*(*i*) ∈ [1, *L*] the chromosome to which window *i* belongs, *L* being the total number of chromosomes (*L* = 16 for *S. cerevisiae*). We also denote by *x*_*i*_ the genomic coordinate of the center of the *i*-th window. Our objective is to infer the genomic coordinates *p* = (*p*_1_, …, *p*_*L*_) of the centromeres of the *L* chromosomes. More precisely, centromeres usually consist of a sequence with a length ranging from several hundred base pairs for point centromeres to several thousand base pairs for regional centromeres. In this work, we infer the mean position of these sequences.

Our main assumption is that, because centromeres colocalize in the nucleus, we expect loci near centromeres in different chromosomes to be enriched in Hi-C contacts. To capture this enrichment, we model the contact counts between windows *i* and *j* of different chromosomes *k* and *l* by a 2-D Gaussian function centered on the corresponding centromeres *p*_*k*_ and *p*_*l*_:

\begin{equation*} a\exp \left(- \frac{(x_i - p_k)^2 + (x_j - p_l)^2)}{2\sigma ^2}\right) + b\,, \end{equation*}

with parameters *a*, *b* and σ ≥ 0. Then, denoting by $\mathcal {D}$ the set of pairs of windows (*i*, *j*) from different chromosomes with non-zero counts, we jointly estimate the parameters (*a*, *b*, σ) and the positions of the *L* centromeres by a least-squares fit of the Hi-C count data, namely, by minimizing in *a*, *b*, σ ≥ 0 and *p* = (*p*_1_, …, *p*_*L*_) the following objective function:

(1) \begin{equation*} \sum _{(i,j)\in \mathcal {D}} \left[ C_{ij} - a\exp \left(- \frac{(x_i - p_{B(i)})^2 + (x_j - p_{B(j)})^2)}{2\sigma ^2}\right) - b \right]^2\,. \end{equation*}

Note that in this optimization, the position of each centromere is constrained to be on its corresponding chromosome. Note also that for each non-zero entry of the contact count matrix, we only fit the Gaussian centered on the corresponding pair of loci. Thus, when the centromeres are close to a chromosome boundary, we only fit a truncated Gaussian.

### Initializing the optimization problem

Because the optimization problem (1) is non convex, the local minimum found by the algorithm depends on the initialization of the parameters, in particular of the centromeres’ positions. We therefore need a heuristic to initialize centromere positions. Because centromeres tend to interact in *trans* with other centromeres, a simple heuristic is to choose the position on each chromosome at the center of the window with the largest total number of *trans* contact counts. However, we found that this heuristic was often not sufficient, because other loci besides centromeres, such as telomeres or rDNA clusters, can exhibit large numbers of *trans* interactions. We therefore implemented another heuristic to generate other good initializations and to explore more local minima. In short, on each chromosome we detect a few local maxima (typically, two per chromosome) of a smoothed *trans* contact counts curve. We then initialize the optimization by combining each choice of centromere location among the candidates on each chromosome. If time constraints do not allow us to test all such initializations (with two choices on 14 chromosomes, this corresponds to 2^14^ = 16 384 different initializations), then we can further reduce the exploration of local minima by starting from the best candidate on each chromosome (i.e. with the largest number of *trans* contact counts), optimizing the objective function from this initialization, and then moving to other ‘nearby’ local minima of the objective function by changing centromere initialization to another candidate one centromere at a time, until no nearby local minimum is better than the one we have converged to.

A Python implementation of the proposed method is available at http://cbio.ensmp.fr/centurion.

### Measuring the performance

To measure the performance of the centromere position prediction on datasets for which we have the ground truth, we compute the distance in base pairs between the prediction *pred* and the segment (*b*, *e*) as follows:

\begin{equation*} \max \left( (b-{\rm pred})_+ , ({\rm pred} - e)_+\right) \end{equation*}

where (*u*)_+_ is *u* if *u* ≥ 0 , 0 otherwise.

## RESULTS

### Validating the method on *S. cerevisiae* and *P. falciparum*

**Figure 1.**
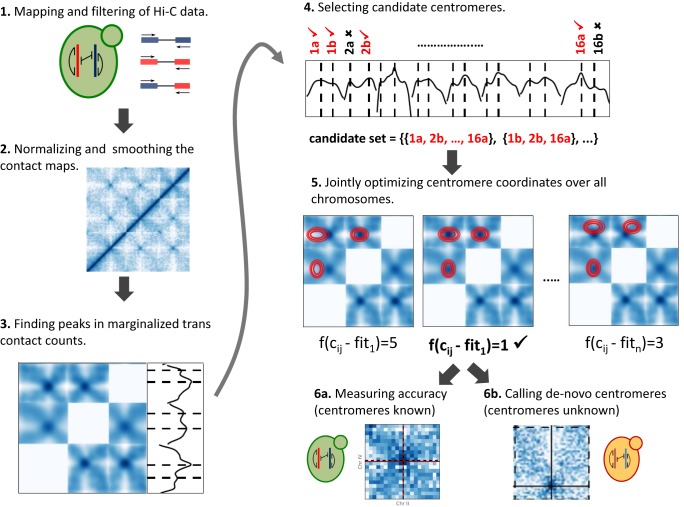
Outline of Centurion's computational workflow. **1**. Paired-end Hi-C reads are mapped and filtered to produce genome-wide contact maps (see Materials and Methods). **2**. Contact maps are normalized to correct for technical and experimental biases (35). **3**. Peaks in marginalized *trans* contact counts are identified as candidate centromere locations. **4**. If necessary, a heuristic reduces the number of centromere candidates that will be used to initialize the joint optimization. **5**. A joint optimization procedure finds the best set of centromere coordinates, one per chromosome, minimizing the squared distance between the 2D Gaussian fits and the observed *trans* contact counts. **6**. For organisms with known centromere locations, the accuracy of predicted centromere locations is evaluated; otherwise, the method provides *de novo* centromere calls.

To evaluate the accuracy of our centromere prediction method (Figure 1), we first applied it to two organisms with known centromere coordinates and available Hi-C data. The first one is the widely studied budding yeast *S. cerevisiae*. The genome of *S. cerevisiae* has 16 chromosomes and thus 16 centromeres, all of which colocalize near the spindle pole body (36). All 32 telomeres of *S. cerevisiae* tether to the nuclear envelope. The repetitive ribosomal DNA of *S. cerevisiae* occurs on chromosome XII and is bundled into the nucleolus at the opposite side of the nucleus from the spindle pole body (37). These organizational principles constrain the chromosomes to fold into a distinct configuration, known as the *Rabl configuration*, which resembles a water lily shape (38). The contacts between centromeres in *S. cerevisiae* chromosomes are known to result in a strong enrichment of centromere-to-centromere Hi-C links (29). We sought to evaluate Centurion's ability to pinpoint the exact centromere locations directly from a Hi-C contact map (39).

**Figure 2.**
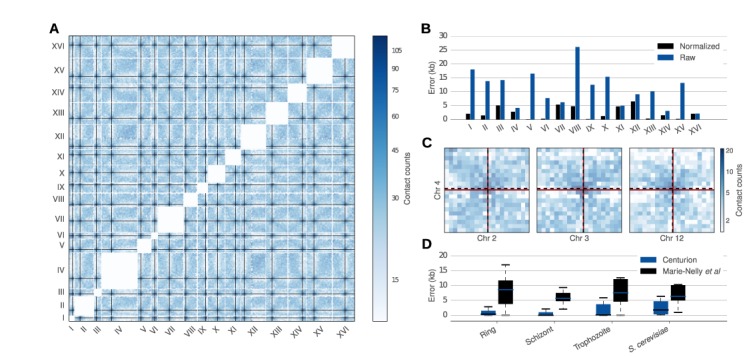
Calling centromeres on *P. falciparum* and *S. cerevisiae*. **A**. Heatmap of the normalized *trans* contact counts for *S. cerevisiae* Hi-C data at 40 kb overlaid with Centurion's centromeres calls (black lines). The contact counts were smoothed with a Gaussian filter (σ = 40 kb) for visualization purposes. White lines indicate chromosome boundaries. **B**. Per chromosome errors of Centurion's centromere calls for *S. cerevisiae* using normalized (black) and raw (blue) Hi-C contact maps at 40 kb resolution. **C**. Heatmap of *trans* contact counts for *P. falciparum* trophozoite data at 40 kb overlaid with Centurion's centromere calls (dashed black line) and ground truth (red line) for chr 2, 3, 4 and 12. **D**. Average errors of centromere calls for Centurion (black) and Marie-Nelly *et al*. (25) method for *S. cerevisiae* data from Duan *et al*. (29) and the three stages of *P. falciparum* when both methods are initialized with the ground truth centromere coordinates.

Using 40 kb-resolution Hi-C contact maps from Duan *et al*. (29) (Figure 2A and B), Centurion predicts centromere coordinates with an average deviation of 11 kb from the known coordinates. Notably, Centurion's Gaussian fitting procedure allows the centromere calls to achieve finer resolution than is provided by the input contact maps. Using 20 kb resolution contact maps, the average deviation drops to 9 kb. Furthermore, we observed that normalizing the contact maps (35) yields substantially improved results, reducing the average deviation to 2.5 kb for both the 20 kb and 40 kb resolution. We investigated the differences in the prediction accuracy of our method among the 16 different chromosomes. While our predictions were within 1 kb of the known centromere coordinates for the chromosomes V, VI, IX, XIII and XV (59 bp, 235 bp, 111 bp, 289 bp and 163 bp away, respectively), they were >5 kb away for chromosomes III, VII and XII (5011 bp, 5327 bp and 6457 bp away, respectively). While the cause of this fluctuation of accuracy is not yet known, chromosomes III and XII house the only major blocks of heterochromatin in this genome other than telomeres (the silent mating loci and rDNA, respectively), suggesting that linked heterochromatinized loci may interfere with accurate centromere prediction.

We then applied our method to a second species, the malaria parasite *P. falciparum*, which is responsible for the most virulent form of malaria (40). We recently used Hi-C to provide a global picture of the genome architecture of *P. falciparum* at three stages (ring, schizont and trophozoite) throughout its erythrocytic life cycle in human blood (26). Centromere coordinates for *P. falciparum* were only identified systematically relatively recently (41). We applied Centurion to the contact maps of each of these three stages at 10 kb, 20 kb and 40 kb resolutions (Supplementary Figure S3). As with *S. cerevisiae*, we observe some variation in the accuracies of our predictions for each chromosome. However, overall, the accuracy is very high. At 10 kb resolution, for example, Centurion's centromere predictions fall within the known centromere location for all 14 chromosomes during the schizont stage, 13 out of 14 for the ring stage and for 11 out of 14 chromosomes in the trophozoite stage. Overall, across the three different stages Centurion correctly localizes 90%, 64% and 45% of centromeres at 10 kb, 20 kb and 40 kb resolution, respectively. For the incorrectly called centromeres, the average distance from Centurion's prediction and the edge of the centromere is 495 bp, 1308 bp and 2319 bp, respectively.

We next sought to understand the sources of error in our predictions. Looking closely at the contact count matrices in the neighborhood of centromeres for which the prediction is not accurate, we observed that loci in proximity to centromeres seem to exhibit unusually sparse interaction counts. For example, Figure 2C shows that in the trophozoite stage, the centromere of chr 1 is close to a chromosome boundary and the chr 4 centromere is close to a locus with few interacting bins. The latter case leads to bias from the normalization procedure because the few non-zero entries in this sparse region are over-corrected. We also investigated whether the accuracy of our prediction varies by life cycle stage and matrix resolution (Supplementary Figure S1). Many chromosomes are given consistently poor centromere calls across all life cycle stages and at all resolutions, corroborating the observations above that the predictions tend to be influenced by biases intrinsic to the genome around those centromeres, such as mappability or GC content.

We next compared the accuracy of our predictions to that of a previously published method (25). The Marie-Nelly *et al*. method often works well for identifying centromeres using Hi-C libraries with very high sequencing depth; however, when Hi-C sequencing depth is limited or when loci other than centromeres strongly cluster, the first step of the procedure, called ‘pre-localization’, sometimes fails to identify the correct fixed size window in which the centromeres reside. We hypothesized that the joint centromere calling by Centurion, which leverages data from all chromosomes at once, might alleviate this instability. To test this hypothesis, we applied the Marie-Nelly *et al*. method to the same four datasets (one *S. cerevisiae* and three *P. falciparum*) described above. As shown in Supplementary Figure S4, in each of these four datasets Centurion identifies centromeres with better accuracy than the Marie-Nelly *et al*. method. For instance, the colocalization of rDNA clusters and virulence genes in *P. falciparum* drastically changes the pattern of the correlation matrix used by Marie-Nelly *et al*. to pre-localize their centromere calls, thus confounding their prediction (Supplementary Figure S5).

We also asked whether the improvement of Centurion over the Marie-Nelly *et al*. method is due to the initialization step, or due to different objective functions used by each method. We initialized both optimization problems with the ground truth and computed the resulting error. Our results (Figure 2D) showed that Centurion's error is still between 4- and 10-fold lower, thus demonstrating the benefit of jointly calling centromeres.

### Resolution, sequencing depth and prediction accuracy

To assess the stability of our predictions, we simulated 500 bootstrapped datasets of *S. cerevisiae* and of each stage of *P. falciparum* with an expected total number of reads equal to the contact count matrices. These bootstrapped samples were obtained by drawing a contact count for each pair of loci i and j from a Poisson distribution of intensity *c*_*ij*_. We then ran the optimization process on the bootstrapped datasets, starting with initial values randomly placed within 40 kb of the centromere calls from our optimization in Supplementary Tables S1–S4. Our results show that the optimization is very stable (average variance of 25 bp for ring, 6 bp for schizont and 12 bp for trophozoite), suggesting that the stochastic sampling of the sequencing procedure does not significantly affect centromere predictions.

We then sought to investigate the extent to which the matrix resolution and sequencing depth affect the accuracy of Centurion's predictions. As already seen in Supplementary Figures S2 and S3, different species give different results: for *S. cerevisiae*, increasing the matrix resolution to 10 kb results in lowered accuracy of centromere calls, while in *P. falciparum* the call quality improves slightly. We speculated that our ability to call centromeres in a given species at a given resolution may depend on the choice of restriction enzyme, the sequencing depth and the resolution of the contact map.

**Figure 3.**
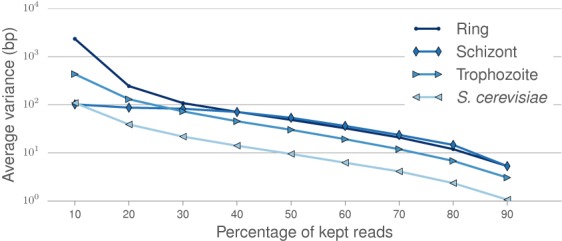
Impact of Hi-C library sequencing depth on the stability of the centromere calls. Average variance of the results of Centurion on 500 generated datasets obtained by downsampling the raw contact counts to the desired coverage.

We next evaluated the effect of depth of sequence coverage on the quality of our centromere predictions. We generated 500 low-coverage datasets by randomly downsampling the raw contact counts. We then ran the optimization process on these downsampled datasets, initializing with perturbed calls as before. We observe that the low-coverage centromere calls remain highly stable and accurate. As illustrated in Figure 3, results across all datasets only begin to degrade when downsampling to <10% of the total number of reads, which corresponds to <1 count per bin on average. Centurion is thus applicable to call centromeres at low cost or for low-abundance species in metagenomic samples.

### Centromere calls on a metagenomic dataset

We next sought to call centromeres in several species simultaneously by combining Centurion with metagenomic Hi-C libraries. We previously (20) generated two Hi-C datasets from synthetic mixtures: one containing 16 yeast strains (including four strains of *S. cerevisiae*), and one containing a mixture of 8 yeasts and 10 prokaryotic species. The two samples contain a total of 19 yeast species, some of which are much better characterized than others: centromere positions are already known for eight species (*K. lactis*, *L. kluyveri*, *L. thermotolerans*, *S. cerevisiae*, *S. kudriavzevii*, *S. mikatae*, *S. pombe*, *S. rouxii*) and partially for one more (*S. bayanus*) (12,42–44).

We aligned the reads from the metagenomic Hi-C datasets to these yeast species’ reference genomes (see Materials and Methods). The quality of the individual species datasets differ greatly because the organisms vary in abundance in the metagenomic samples, and because many sequences are shared nearly identically between organisms, making the number of uniquely mappable reads for each organism range between 109 k for one of the *S. cerevisiae* strains and 26 M for the bacteria *V. fischeri*. Consequently, the sparsity of the matrices is variable (Supplementary Tables S5 and S6). Furthermore, some contact count matrices include at least one interaction count for >99% of all possible locus pairs, whereas other matrices are below 5%. Similarly, in the 40 kb matrices, the average number of interchromosomal contact counts per bin varies from <0.004 to >200. In particular, the matrices for the four *S. cerevisiae* strains are very sparse: the reference genomes of the four strains are very similar to one another; thus, we are not able to map reads uniquely. We therefore discarded those strains from our analysis, as well as organisms with incomplete reference genomes. We applied Centurion to the remaining 14 yeasts (*E. gossypii*, *K. lactis*, *K. wickerhamii*, *L. kluyveri*, *L. waltii*, *S. bayanus*, *S. kudriavzevii*, *S. mikatae*, *S. paradoxus*, *S. stipitis*, *P. pastoris*, *L. thermotolerans*, *S. pombe*, *S. rouxii*) on both 20 kb and 40 kb contact maps.

**Figure 4.**
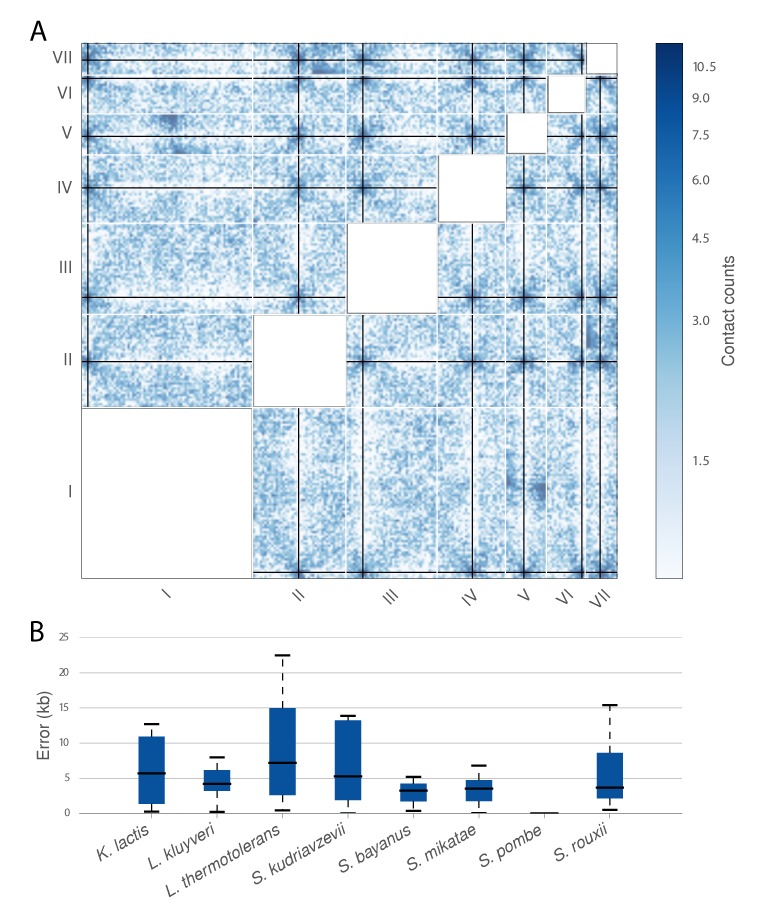
Centromere calling on a metagenomic sample. **A**. Heatmap of the *trans* contact counts for *K. wickerhamii* overlaid with *de novo* centromere calls (black lines). The contact counts were smoothed with a Gaussian filter (σ = 40 kb) for visualization purposes. White lines indicate chromosome boundaries. **B**. Box plots indicating the error (in kb) for each chromosome in Centurion's centromere calls for eight yeasts with known centromere coordinates from the combined metagenomic Hi-C samples M-3D and M-Y of (20) on the 20 kb contact count matrices.

Across these 14 species Centurion performs well, both on high-coverage datasets (*K. lactis*, *L. kluyveri*, *S. bayanus*) and low-coverage datasets (*S. mikatae*), at 20 kb and 40 kb, finding centromeres at an average deviation from the ground truth of 10 kbp (Figure 4B and Supplementary Figure S6). Given this success with yeasts with known centromere positions, we next made *de novo* centromere calls for the other six yeast species present in the metagenomic samples. These regions, visualized in Supplementary Figures S15–S20, are strong candidates for experimental validation by other approaches. One feature that is shared by centromeres across all studied fungi is that they reside in regions of early replication timing (3,4). Thus if our centromere calls lie in regions of advanced replication timing in a species for which replication timing has been profiled but centromeres have not yet been identified, this data could be used to assess the validity of our predictions. Accordingly, we overlaid the positions of our centromere calls in *P. pastoris*, where replication has been recently profiled (45). In all four chromosomes, *P. pastoris* centromere predictions lay in regions of early replication timing (Supplementary Figure S21), lending support to our predictions.

### The effect of the choice of restriction enzyme

In addition to the resolution of our contact matrices, the underlying resolution of the Hi-C data itself may limit the accuracy of our predictions. Hi-C reads can only occur near the recognition site of the restriction enzyme used in the Hi-C assay; indeed, the best resolution we can hope to achieve is a matrix in which each corresponds to one restriction enzyme fragment. Some restriction enzymes cut much more frequently than others. Thus, we speculated that a Hi-C experiment using enzymes that cut more frequently might yield more accurate results than an experiment using less frequently cutting enzymes.

To address this question, we compare the accuracy of centromere calling from two Hi-C libraries created from a single metagenomic sample using different combinations of restriction enzymes. The first library was created using the two 6 bp-cutters, HindIII and NcoI. The second library uses Sau3AI, which has a 4 bp recognition site, and AflIII, which has a 6 bp recognition site with two degenerate sites, making it effectively a 5 bp cutter. Digestion with HindIII/NcoI yields a total of 8324 restriction fragments, whereas digestion with Sau3AI/AflIII yields 42359 restriction fragments. We corrected for the difference in Hi-C sequencing depth between Sau3AI/AflIII and the NcoI/HindIII libraries by generating downsampled datasets with an equal number of reads from each sequencing library. We then normalized the datasets and applied Centurion. The sample includes three species for which we possess the ground truth centromere locations, only one of which (*L. thermotolerans*) had enough reads in both the Ncol/HindIII (63 000 reads) and the pooled Sau3AI/AflIII (55 000 reads) datasets to correctly call the centromeres. The error on the downsampled Sau3AI/AflIII datasets (8 kbp) was on average half as large as the error on the the Ncol/HindIII datasets (16 kbp). Thus, we conclude that using a restriction enzyme with more frequent cutting sites enables more precise centromere calls at fine scales.

## DISCUSSION

While centromeres are a fundamental element in the biology of genomes, their identification in diverse species has proven difficult due to sequence divergence and limitations of available tools. In this work, we have developed a novel method, Centurion, that uses centromere colocalization and the pattern it creates in Hi-C contact maps to jointly call centromeres for all chromosomes of an organism. We first established the feasibility of this approach by demonstrating that Centurion accurately calls regional centromeres on the parasite *P. falciparum* and the yeast *S. cerevisiae* as well as point centromeres on several other yeasts with known centromere coordinates. For the species with high depth Hi-C sequencing, Centurion often identified centromeres within 1 kb of the actual coordinates (41 times out of 58 for three stages of *P. falciparum* and *S. cerevisiae* data). We then used Centurion to infer centromeres of multiple yeast species (eight with known, six with unknown centromere coordinates) from two metagenomic Hi-C samples. Our results showed that Centurion still accurately identifies centromere coordinates from samples with only limited sequencing depth. Thus, Centurion can be used to accurately and efficiently identify centromere locations in yeast species.

The task of centromere identification from Hi-C data has been attempted recently by others (25). Centurion offers a few key differences compared to the previous approach. The first difference is in the pre-localization of candidate centromeres. Marie-Nelly *et al*.'s method uses only the *cis* Pearson correlation information independently per chromosome to identify the initial candidates. However, the pattern created by centromeres in the Pearson correlation matrix can be very similar to the patterns generated by other genomic elements such as rDNA coding regions or by specific gene clusters (e.g. virulence genes in *P. falciparum*). Because Marie-Nelly *et al*.'s method restricts the further search for the best centromere coordinate to only the candidates from the pre-localization step, an inaccurate candidate (e.g. an rDNA region instead of a centromere) will prevent the method from finding the correct centromere location. Centurion, on the other hand, utilizes *trans* contact information jointly across all chromosomes for its pre-localization step. Furthermore, Centurion allows multiple candidates per chromosome during the second step of the optimization, thereby leaving room for correcting potential errors in the pre-localization step. The second difference between the two methods is in how they use the submatrices that correspond to *trans* contact maps flanking the pairs of candidate centromeres from the pre-localization step. For an organism with N chromosomes, Marie-Nelly *et al*.'s method carves out the N-1 *trans* submatrices for each chromosome, sums these N-1 matrices and then collapses the sum into a 1D vector of row/column sums. Then, independently for each chromosome, the method fits a Gaussian to this 1D vector, and the resulting peak corresponds to the predicted centromere location. In this procedure, both the summation of N-1 matrices and the collapsing of the resulting matrix into a 1D vector of sums result in loss of important information embedded in 2D maps. Furthermore, performing the Gaussian fit separately for each chromosome does not fully take into account the joint colocalization of the other N-1 centromeres. To address these issues, Centurion infers a 2D Gaussian fit that best explains the observed *trans* contact counts, jointly optimizing these 2D fits for all pairs of centromeres. Both of these improvements in the pre-localization and the optimization steps allow Centurion to perform better specifically for the cases with limited sequencing depth.

Our approach could be improved in several respects. First, better modeling of zero contact counts may improve inference for organisms with many repeated sequences in the pericentromeric regions, or datasets with low sequencing depth. Second, one could model contact counts as a Gaussian distribution centered on the pairs of centromere locations. Maximizing the log likelihood of such a model might yield improved performance. Last, as described here, our method requires reference genomes for the metagenomic samples. It would be possible to first build reference genomes directly from the Hi-C data, using methods like Lachesis (17) or GRAAL (46), and then infer centromere locations using the inferred references. However, the inherent structure of Hi-C contact counts for organisms with colocalizing centromeres will likely present a challenge for these methods because pericentromeric sequences on different chromosomes are likely to appear to be adjacent to one another.

Finally, our new centromere predictions have practical applications. Autonomously replicating plasmids and artificial chromosomes are useful tools for research and strain engineering (9). Identification of centromeres in new species will facilitate building such constructs over an expanded species range. *P. pastoris*, for example, is a common industrial chassis (47), but existing plasmid tools in the species have elevated loss rates (48) that could be stabilized by addition of a centromere. Many of our centromere calls were accurate to <1 kb, making experimental validation possible. Sequencing data is available from the Short Read Archive at http://www.ncbi.nlm.nih.gov/Traces/sra/?study=SRP057812.

## SUPPLEMENTARY DATA

Supplementary Data are available at NAR Online.

SUPPLEMENTARY DATA
