# Supplementary material for: Accurate identification of centromere locations in yeast genomes using Hi-C
Source: Nucleic Acids Res. 2015 May 4;43(11):5331–9. doi: 10.1093/nar/gkv424 (PMC4477656; doi:10.1093/nar/gkv424)
Supplement: SUPPLEMENTARY DATA [file supp_43_11_5331__index.html]

Accurate identification of centromere locations in yeast genomes using Hi-C — Accurate identification of centromere locations in yeast genomes using Hi-C — SUPPLEMENTARY DATA 

# Accurate identification of centromere locations in yeast genomes using Hi-C

## SUPPLEMENTARY DATA

**Files in this Data Supplement:**

- SUPPLEMENTARY DATA
