# Supplementary material for: Accurate identification of centromere locations in yeast genomes using Hi-C
Source: Nucleic Acids Res. 2015 May 4;43(11):5331–9. doi: 10.1093/nar/gkv424 (PMC4477656; doi:10.1093/nar/gkv424)
Supplement: SUPPLEMENTARY DATA [file supp_gkv424_nar-03671-n-2014-File014.pdf]

Supplementary Information

March 24, 2015

List of Tables

1 **Centromere calls for *S. cerevisiae*, ground truth and errors** 6
2 **Centromere calls for *P. falciparum* (ring stage), ground truth and errors** . . 6
3 **Centromere calls for *P. falciparum* (trophozoite stage), ground truth and errors** 7
4 **Centromere calls for *P. falciparum* (schizont stage), ground truth and errors** 7
5 **Centromere calls for *A. thaliana*, annotation units and errors** 8
6 **M-3D multi-sample statistics for each organism's contact counts matrices (20 kb)** 10
7 **M-Y multi-sample statistics for each organism's contact counts matrices (20 kb)** 11
8 ***K. lactis* centromere calls, ground truth and errors** 13
9 ***L. kluyveri* centromere calls, ground truth and errors** 14
10 ***S. bayanus* centromere calls, partial ground truth and errors** 15
11 ***S. mikatae* centromere calls, ground truth and errors** 16
12 ***S. kudriavzevii* centromere calls, ground truth and errors** 17
13 ***L. thermotolerans* centromere calls, ground truth and errors** 18
14 ***S. pombe* centromere calls, ground truth and errors** 19
15 ***Z. rouxii* centromere calls, ground truth and errors** 20
16 ***P. pastoris* de novo centromere calls** 21
17 ***E. gossypii* de novo centromere calls** 22
18 ***K. wickerhamii* de novo centromere calls** 23
19 ***L. waltii* de novo centromere calls** 24
20 ***S. paradoxus* de novo centromere calls** 25
21 ***S. stipitis* de novo centromere calls** 26

List of Figures

1 **Error on centromere calls for *P. falciparum* on raw and normalized contact counts (40 kb)** 3
2 **Error on centromere calls for *S. cerevisiae* at different resolutions (10 kb, 20 kb, 40 kb)** 4

3 **Error on centromere calls for *P. falciparum* at different resolutions (10 kb, 20 kb, 40 kb)** . . . 5
4 **Centurion vs Marie-Nelly et al. [2014]'s method** . . . 9
5 **Pearson correlation matrix of *P. falciparum*'s chr XII.** . . . 9
6 **Errors on metagenomic sample** . . . 12
7 **Centromere calls for *K. lactis*** . . . 13
8 **Centromere calls for *L. kluyveri*** . . . 14
9 **Centromere calls for *S. bayanus*** . . . 15
10 **Centromere calls for *S. mikatae*** . . . 16
11 **Centromere calls for *S. kudriavzevii*** . . . 17
12 **Centromere calls for *L. thermotolerans*** . . . 18
13 **Centromere calls for *S. pombe*** . . . 19
14 **Centromere calls for *Z. rouxii*** . . . 20
15 **Centromere calls for *P. pastoris*** . . . 21
16 **Centromere calls for *E. gossypii*** . . . 22
17 **Centromere calls for *K. wickerhamii*** . . . 23
18 **Centromere calls for *L. waltii*** . . . 24
19 **Centromere calls for *S. paradoxus*** . . . 25
20 **Centromere calls for *S. stipitis*** . . . 26
21 **Replication timing profile across the *P. pastoris* genome** . . . 27

Contents

Supplementary Figure 1: Error on centromere calls for *P. falciparum* on raw and normalized contact counts (40 kb)

A. ring stage **B.** trophozoite stage **C.** schizont stage

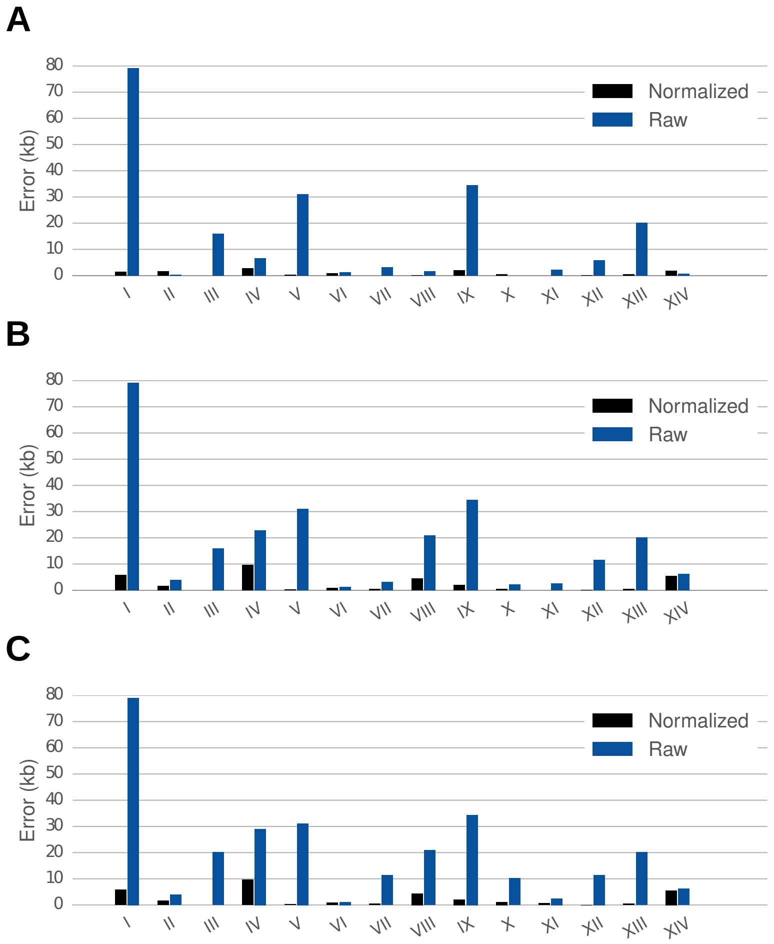

Supplementary Figure 2: **Error on centromere calls for *S. cerevisiae* at different resolutions (10 kb, 20 kb, 40 kb)**

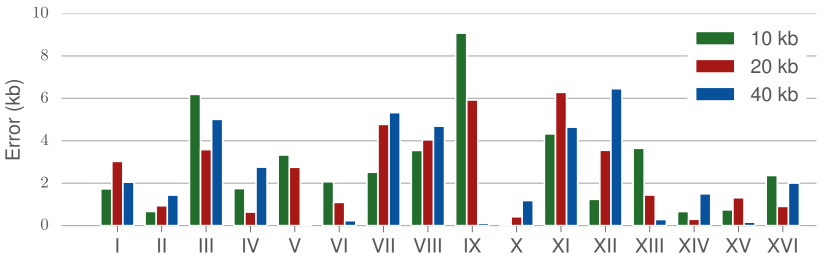

Supplementary Figure 3: **Error on centromere calls for *P. falciparum* at different resolutions (10 kb, 20 kb, 40 kb)**

A. ring stage **B.** trophozoite stage **C.** schizont stage

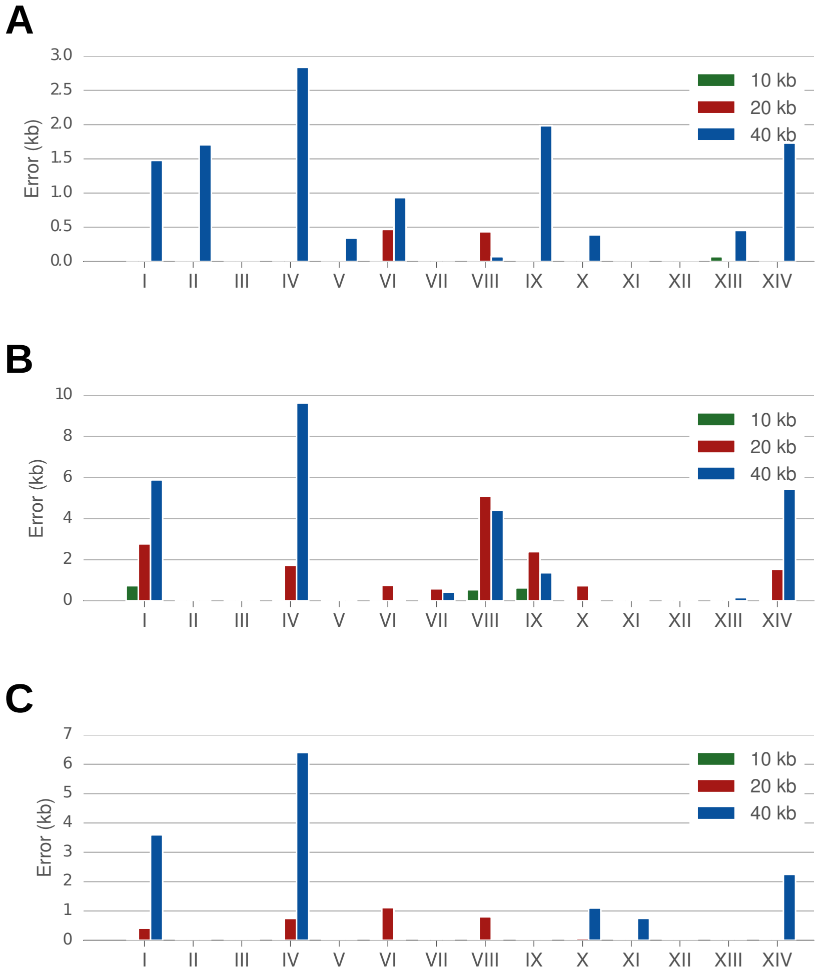

Supplementary Table 1: **Centromere calls for *S. cerevisiae*, ground truth and errors**

Chromosome	Ground truth	10 kb		20 kb		40 kb	
		Call	Error	Call	Error	Call	Error
I	151 584 - 151 584	153 319	1735	154 614	3030	153 633	2049
II	238 325 - 238 325	238 994	669	237 386	939	236 883	1442
III	114 499 - 114 499	108 309	6190	110 914	3585	109 488	5011
IV	449 819 - 449 819	451 567	1748	450 459	640	452 579	2760
V	152 103 - 152 103	155 434	3331	149 350	2753	152 162	59
VI	148 622 - 148 622	150 691	2069	149 718	1096	148 387	235
VII	497 042 - 497 042	499 561	2519	501 816	4774	502 369	5327
VIII	105 698 - 105 698	102 152	3546	101 652	4046	101 007	4691
IX	355 742 - 355 742	364 818	9076	361 667	5925	355 631	111
X	436 418 - 436 418	436 467	49	435 999	419	437 603	1185
XI	439 889 - 439 889	444 216	4327	446 174	6285	444 533	4644
XII	150 946 - 150 946	149 704	1242	147 393	3553	144 489	6457
XIII	268 149 - 268 149	264 502	3647	266 704	1445	267 860	289
XIV	628 877 - 628 877	629 542	665	629 178	301	627 374	1503
XV	326 703 - 326 703	327 448	745	328 019	1316	326 866	163
XVI	556 070 - 556 070	553 705	2365	555 162	908	554 062	2008

Supplementary Table 2: **Centromere calls for *P. falciparum* (ring stage), ground truth and errors**

Chromosome	Ground truth	10 kb		20 kb		40 kb	
		Call	Error	Call	Error	Call	Error
I	456 871 - 461 511	458 108	0	457 710	0	455 394	1477
II	446 771 - 450 941	448 688	0	448 953	0	452 647	1706
III	597 014 - 601 275	599 187	0	597 486	0	599 779	0
IV	641 019 - 645 339	644 178	0	644 931	0	648 176	2837
V	454 543 - 458 793	456 329	0	455 929	0	454 201	342
VI	477 756 - 482 016	480 602	0	477 287	469	482 950	934
VII	808 365 - 812 875	811 744	0	810 236	0	812 304	0
VIII	297 895 - 302 515	299 983	0	297 460	435	297 824	71
IX	1 241 081 - 1 245 451	1 242 788	0	1 242 570	0	1 247 435	1984
X	935 682 - 937 823	937 162	0	936 247	0	938 213	390
XI	830 782 - 835 432	832 728	0	832 858	0	834 051	0
XII	1 281 521 - 1 285 941	1 284 567	0	1 285 214	0	1 285 943	2
XIII	1 167 070 - 1 171 720	1 166 999	71	1 168 375	0	1 172 174	454
XIV	1 070 909 - 1 075 369	1 072 595	0	1 072 131	0	1 069 179	1730

Supplementary Table 3: **Centromere calls for *P. falciparum* (trophozoite stage), ground truth and errors**

Chromosome	Ground truth	10 kb		20 kb		40 kb	
		Call	Error	Call	Error	Call	Error
I	456 871 - 461 511	456 134	737	454 096	2775	450 980	5891
II	446 771 - 450 941	448 623	0	447 562	0	448 953	0
III	597 014 - 601 275	598 035	0	597 348	0	597 426	0
IV	641 019 - 645 339	645 248	0	647 059	1720	654 977	9638
V	454 543 - 458 793	455 899	0	455 305	0	457 291	0
VI	477 756 - 482 016	480 552	0	477 010	746	480 417	0
VII	808 365 - 812 875	810 348	0	807 779	586	807 937	428
VIII	297 895 - 302 515	297 355	540	292 808	5087	293 495	4400
IX	1 241 081 - 1 245 451	1 240 449	632	1 238 687	2394	1 239 714	1367
X	935 682 - 937 823	936 765	0	938 559	736	937 531	0
XI	830 782 - 835 432	832 425	0	833 938	0	833 994	0
XII	1 281 521 - 1 285 941	1 284 634	0	1 284 403	0	1 282 674	0
XIII	1 167 070 - 1 171 720	1 168 647	0	1 168 225	0	1 166 916	154
XIV	1 070 909 - 1 075 369	1 071 170	0	1 069 381	1528	1 065 476	5433

Supplementary Table 4: **Centromere calls for *P. falciparum* (schizont stage), ground truth and errors**

Chromosome	Ground truth	10 kb		20 kb		40 kb	
		Call	Error	Call	Error	Call	Error
I	456 871 - 461 511	458 269	0	456 457	414	453 275	3596
II	446 771 - 450 941	448 913	0	448 374	0	450 640	0
III	597 014 - 601 275	599 091	0	597 694	0	598 616	0
IV	641 019 - 645 339	644 640	0	646 085	746	651 741	6402
V	454 543 - 458 793	455 379	0	454 880	0	455 537	0
VI	477 756 - 482 016	479 786	0	476 643	1113	479 443	0
VII	808 365 - 812 875	810 510	0	809 034	0	810 833	0
VIII	297 895 - 302 515	299 463	0	297 094	801	299 394	0
IX	1 241 081 - 1 245 451	1 242 617	0	1 242 663	0	1 244 982	0
X	935 682 - 937 823	936 637	0	935 616	66	934 581	1101
XI	830 782 - 835 432	832 307	0	831 740	0	830 033	749
XII	1 281 521 - 1 285 941	1 284 123	0	1 284 309	0	1 283 900	0
XIII	1 167 070 - 1 171 720	1 168 687	0	1 169 725	0	1 171 143	0
XIV	1 070 909 - 1 075 369	1 072 384	0	1 071 648	0	1 068 660	2249

Supplementary Table 5: Centromere calls for *A. thaliana*, annotation units and errors

Chromosome	Ground truth	40 kb	
		Call	Error
1	15 086 046 - 15 087 045	15 047 165	39 380
2	3 607 930 - 3 608 929	3 841 087	232 657
3	14 132 042 - 14 208 952	14 177 317	6820
4	3 956 022 - 3 957 021	3 754 384	202 137
5	11 725 025 - 11 726 024	12 055 189	329 665

Supplementary Figure 4: Centurion vs Marie-Nelly et al. [2014]'s method

Centurion and Marie-Nelly et al. [2014]'s whole pipeline centromere calls error on 40 kb contact counts matrices. Marie-Nelly et al. [2014]'s method fails to prelocalize properly centromeres.

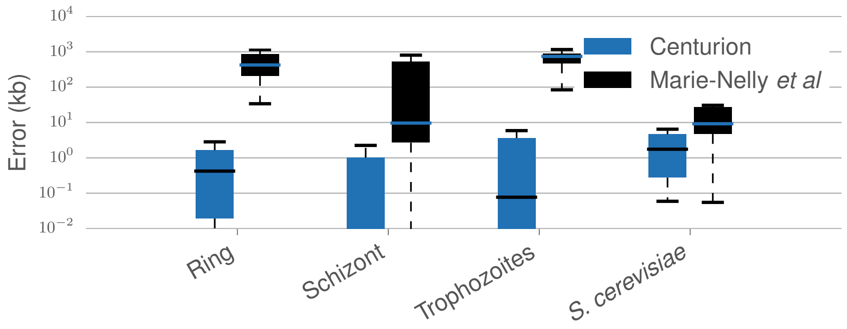

Supplementary Figure 5: Pearson correlation matrix of *P. falciparum*'s chr XII.

Dashed black line indicates the centromere. Because var genes strongly colocalize, the typical X-shape found in *S. cerevisiae*'s Pearson correlation matrices completely disappears, consequently causing Marie-Nelly et al. [2014]'s first step to fail to prelocalize centromeres.

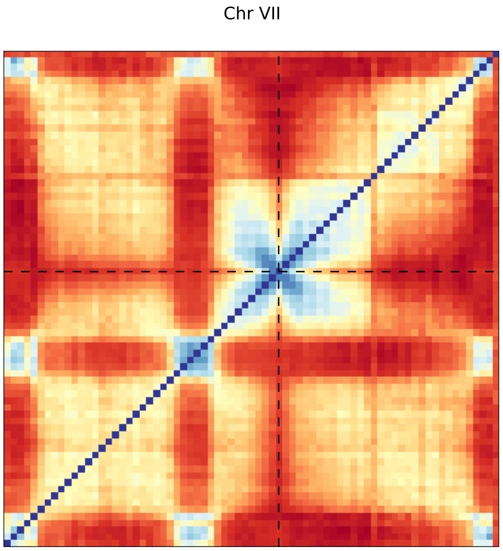

Supplementary Table 6: **M-3D multi-sample statistics for each organism's contact counts matrices (20 kb)**

For each contact count matrix, we compute several statistics: (1) the average number of contact counts off-diagonal, (2) the percentage of non-zero element off-diagonal, (3) the average number of *trans* contact counts, (4) the percentage of non-zero *trans* contact counts

**Organism**	**Number of chrom**	**Average contact counts per bin**	**sparsity**	**Average *trans* contact counts per bin**	***trans* sparsity**
Acinetobacter sp. ADP1	1	91.11	99.44	-	-
Vibrio fischeri ES114	2	87.14	99.53	57.23	100.00
Methanococcus maripaludis	2	82.29	96.47	0.00	0.00
Burkholderia thailandensis E264	2	37.52	99.70	27.92	100.00
Escherichia coli str. K-12 substr. DH10B	1	35.79	90.39	-	-
Flavobacterium johnsoniae UW101	1	30.88	99.55	-	-
Rhodopseudomonas palustris CGA009	1	30.58	99.61	-	-
Bacillus subtilis subsp. subtilis str. 168	1	13.53	99.21	-	-
Schizosaccharomyces pombe	3	0.91	32.15	0.35	24.38
Pichia pastoris GS115	4	0.72	32.47	0.39	28.06
Zygosaccharomyces rouxii strain CBS732	7	0.52	24.97	0.28	20.80
Kluyveromyces thermotolerans strain CBS6340	8	0.48	23.24	0.27	20.25
Saccharomyces cerevisiae S288c	16	0.28	16.01	0.18	14.32
Pseudomonas fluorescens Pf0-1	1	0.02	1.35	-	-

Supplementary Table 7: M-Y multi-sample statistics for each organism's contact counts matrices (20 kb)

For each contact count matrix, we compute several statistics: (1) the average number of contact counts off-diagonal, (2) the percentage of non-zero element off-diagonal, (3) the average number of *trans* contact counts, (4) the percentage of non-zero *trans* contact counts

**Organism**	**Number of chrom**	**Average contact counts per bin**	**sparsity**	**Average *trans* contact counts per bin**	***trans* sparsity**
Kluyveromyces lactis	6	3.20	74.45	1.85	72.64
Lachancea kluyveri	8	2.83	67.70	1.49	65.44
Lachancea waltii	8	2.38	60.39	1.22	57.89
Kluyveromyces wickerhamii	7	1.43	45.38	0.66	41.49
Scheffersomyces stipitis	8	1.38	45.08	0.72	42.01
Saccharomyces mikatae	16	1.35	48.58	0.82	46.65
Saccharomyces bayanus	16	0.94	35.49	0.51	33.19
Saccharomyces paradoxus	16	0.69	26.57	0.35	24.46
Pichia pastoris GS115	4	0.37	25.48	0.28	23.71
Eremothecium gossypii	7	0.13	7.06	0.06	4.82
Saccharomyces kudriavzevii	16	0.11	5.98	0.05	4.73
Saccharomyces cerevisiae SK1	16	0.02	0.92	0.01	0.65
Saccharomyces cerevisiae S288c	16	0.00	0.11	0.00	0.07

Supplementary Figure 6: **Errors on metagenomic sample**

Box plots indicating the error (in kb) for each chromosome in Centurion's centromere calls for eight yeasts with known centromere coordinates from the combined metagenomic Hi-C samples M-3D and M-Y on the 40 kb contact count matrices.

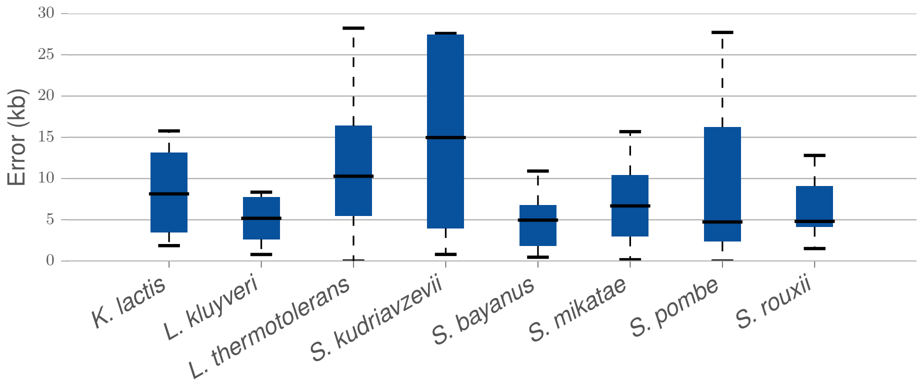

Supplementary Figure 7: **Centromere calls for *K. lactis***

Smoothed *trans* contact counts (with $\sigma = 40$ kb) overlaid with Centurion's centromere calls (black line). White lines represent chromosome boundaries.

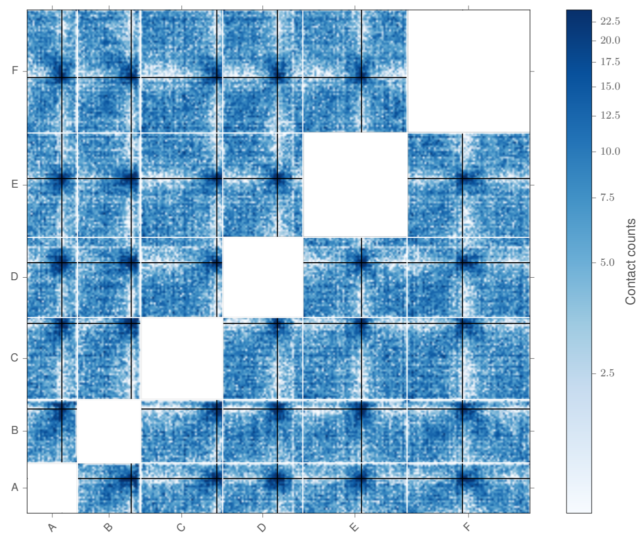

Supplementary Table 8: ***K. lactis* centromere calls, ground truth and errors**

Chromosome	Ground truth	20 kb		40 kb	
		Call	Error	Call	Error
A	760 404 - 760 598	747 703	12 701	744 213	16 191
B	1 168 861 - 1 169 058	1 156 659	12 202	1 155 652	13 209
C	1 638 151 - 1 638 347	1 633 885	4266	1 632 850	5301
D	1 187 303 - 1 187 500	1 180 157	7146	1 174 906	12 397
E	1 263 806 - 1 264 001	1 264 257	256	1 260 994	2812
F	1 187 015 - 1 187 211	1 186 655	360	1 189 411	2200

Supplementary Figure 8: Centromere calls for *L. kluyveri*

Smoothed *trans* contact counts (with σ = 40 kb) overlaid with Centurion's centromere calls (black line). White lines represent chromosome boundaries.

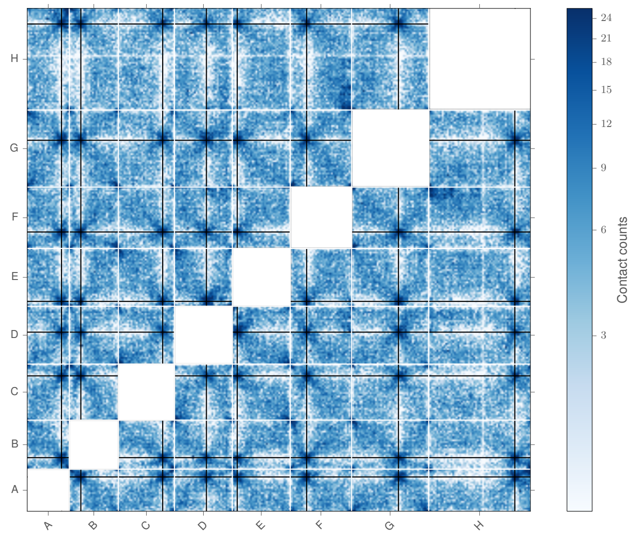

Supplementary Table 9: *L. kluyveri* centromere calls, ground truth and errors

Chromosome	Ground truth	20 kb		40 kb	
		Call	Error	Call	Error
A	777 082 - 777 277	784 872	7595	782 908	5631
B	272 171 - 272 366	268 270	3901	263 855	8316
C	1 009 526 - 1 009 330	1 008 047	1479	1 011 248	1918
D	737 092 - 737 289	729 108	7984	728 743	8349
E	108 420 - 108 235	113 926	5691	111 117	2882
F	383 306 - 383 110	378 812	4494	375 717	7589
G	1 064 569 - 1 064 371	1 068 157	3786	1 069 100	4729
H	1 963 796 - 1 963 599	1 963 570	226	1 964 393	794

Supplementary Figure 9: Centromere calls for *S. bayanus*

Smoothed *trans* contact counts (with $\sigma = 40$ kb) overlaid with Centurion's centromere calls (black line). White lines represent chromosome boundaries.

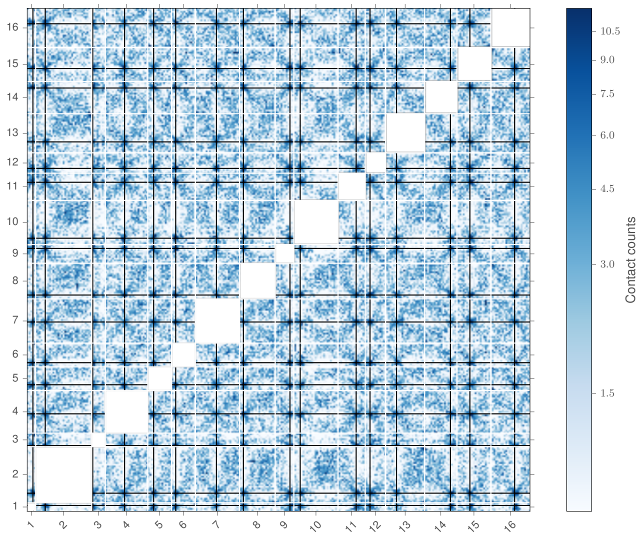

Supplementary Table 10: *S. bayanus* centromere calls, partial ground truth and errors

Chromosome	Ground truth	20 kb		40 kb	
		Call	Error	Call	Error
1	128 493 - 128 610	133 793	5183	135 493	6883
2	-	227 362	-	225 392	-
3	24 732 - 24 851	24 374	358	24 672	60
4	447 057 - 447 177	449 935	2758	453 979	6802
5	127 728 - 127 885	131 258	3373	138 605	10 720
6	-	107 819	-	97 831	-
7	-	490 402	-	496 199	-
8	102 036 - 102 155	101 405	631	91 014	11 022
9	342 506 - 342 624	345 821	3197	348 290	5666
10	-	151 519	-	148 837	-
11	424 482 - 424 587	424 027	455	426 525	1938
12	-	113 924	-	113 109	-
13	258 015 - 258 136	253 649	4366	257 327	688
14	609 003 - 609 122	604 920	4083	605 657	3346
15	301 003 - 301 123	305 724	4601	304 941	3818
16	560 121 - 560 240	556 888	3233	558 583	1538

Supplementary Figure 10: **Centromere calls for *S. mikatae***

Smoothed *trans* contact counts (with $\sigma = 40$ kb) overlaid with Centurion's centromere calls (black line). White lines represent chromosome boundaries.

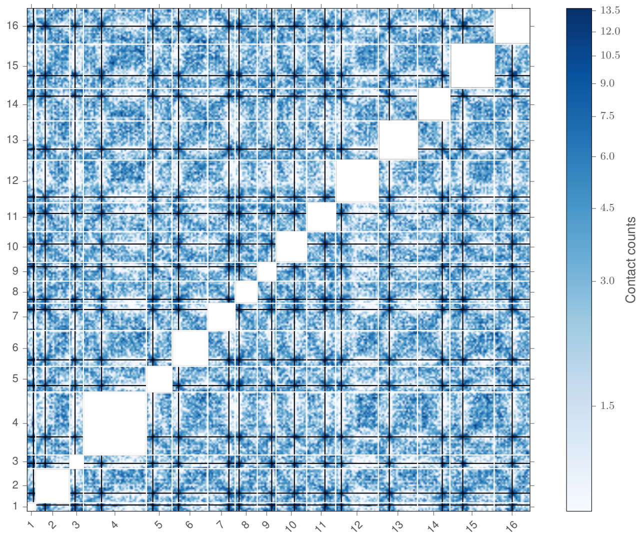

Supplementary Table 11: ***S. mikatae* centromere calls, ground truth and errors**

Chromosome	Ground truth	20 kb		40 kb	
		Call	Error	Call	Error
1	134 639 - 134 759	139 227	4468	147 416	12 657
2	225 947 - 226 062	226 196	134	223 853	2094
3	112 549 - 112 682	123 596	10 914	123 147	10 465
4	428 996 - 429 115	425 527	3469	425 807	3189
5	155 935 - 156 053	152 599	3336	148 911	7024
6	155 876 - 155 995	156 306	311	152 732	3144
7	488 820 - 488 935	491 034	2099	493 846	4911
8	84 409 - 84 527	90 055	5528	92 846	8319
9	331 647 - 331 767	335 329	3562	339 195	7428
10	433 451 - 433 569	429 847	3604	430 766	2685
11	426 605 - 426 749	430 382	3633	428 157	1408
12	137 938 - 138 058	134 774	3164	133 389	4549
13	259 194 - 259 326	259 361	35	258 359	835
14	587 008 - 587 124	580 201	6807	581 480	5528
15	293 868 - 293 999	294 747	748	302 652	8653
16	432 872 - 432 990	419 838	13 034	422 199	10 673

Supplementary Figure 11: **Centromere calls for *S. kudriavzevii***

Smoothed *trans* contact counts (with $\sigma = 40$ kb) overlaid with Centurion's centromere calls (black line). White lines represent chromosome boundaries.

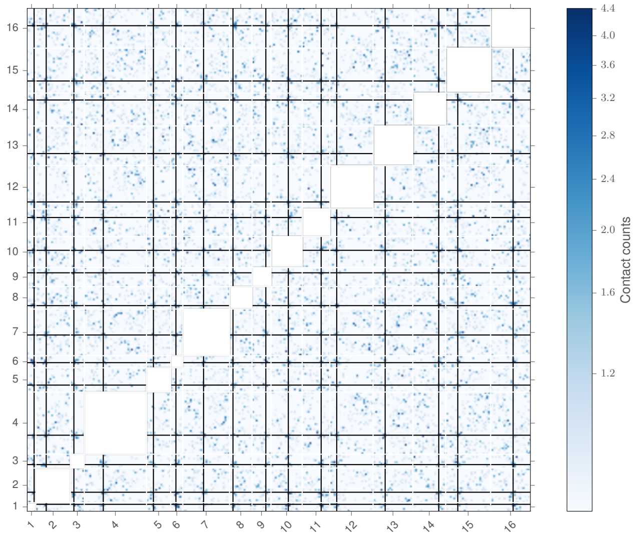

Supplementary Table 12: ***S. kudriavzevii* centromere calls, ground truth and errors**

Chromosome	Ground truth	20 kb		40 kb	
		Call	Error	Call	Error
1	126 503 - 126 621	139 821	13 200	159 827	33 206
2	218 375 - 218 494	280 091	61 597	239 855	21 361
3	93 380 - 93 501	79 993	13 387	80 093	13 287
4	441 296 - 441 418	440 008	1288	439 893	1403
5	148 755 - 148 877	220 186	71 309	159 993	11 116
6	144 259 - 144 379	140 074	4185	120 144	24 115
7	499 997 - 500 118	500 033	0	480 114	19 883
8	87 050 - 87 170	80 118	6932	80 113	6937
9	326 489 - 326 613	320 133	6356	320 091	6398
10	403 891 - 404 009	399 992	3899	400 093	3798
11	421 054 - 421 176	420 045	1009	439 781	18 605
12	142 068 - 142 189	139 973	2095	159 948	17 759
13	253 924 - 254 043	240 052	13 872	279 914	25 871
14	595 631 - 595 753	599 954	4201	599 937	4184
15	286 560 - 286 681	279 996	6564	280 098	6462
16	520 694 - 520 812	519 870	824	519 977	717

Supplementary Figure 12: **Centromere calls for *L. thermotolerans***

Smoothed *trans* contact counts (with $\sigma = 40$ kb) overlaid with Centurion's centromere calls (black line). White lines represent chromosome boundaries.

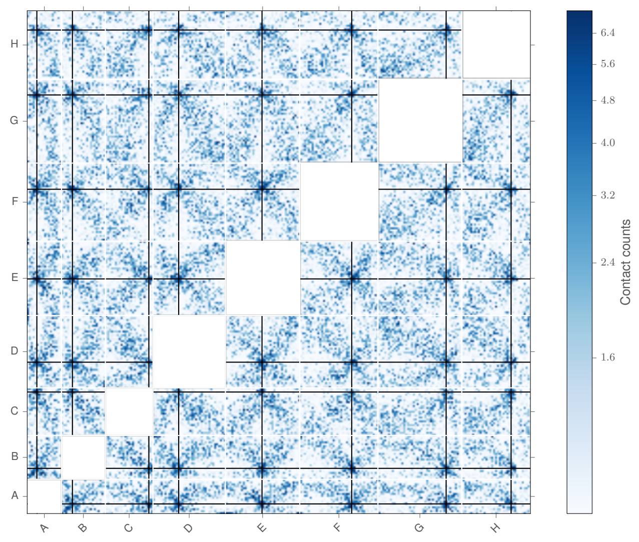

Supplementary Table 13: ***L. thermotolerans*** **centromere calls, ground truth and errors**

Chromosome	Ground truth	20 kb		40 kb	
		Call	Error	Call	Error
A	186 515 - 186 379	201 225	14 846	202 706	16 327
B	238 312 - 238 187	235 631	2681	229 282	9030
C	912 837 - 912 964	920 560	7596	912 875	0
D	555 337 - 555 463	553 006	2331	538 813	16 524
E	761 047 - 760 921	767 727	6806	767 725	6804
F	1 078 717 - 1 078 842	1 094 222	15 380	1 090 376	11 534
G	1 432 769 - 1 432 902	1 432 357	412	1 431 366	1403
H	1 062 917 - 1 063 043	1 040 425	22 492	1 034 688	28 229

Supplementary Figure 13: **Centromere calls for *S. pombe***

Smoothed *trans* contact counts (with $\sigma = 40$ kb) overlaid with Centurion's centromere calls (black line). White lines represent chromosome boundaries.

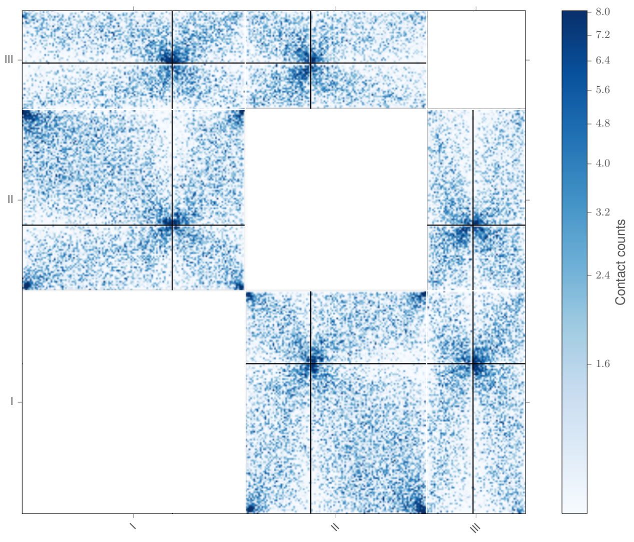

Supplementary Table 14: ***S. pombe* centromere calls, ground truth and errors**

Chromosome	Ground truth	20 kb		40 kb	
		Call	Error	Call	Error
I	3 753 687 - 3 789 421	3 764 436	0	3 767 270	0
II	1 602 264 - 1 644 747	1 619 912	0	1 649 483	4736
III	1 070 904 - 1 137 003	1 121 329	0	1 164 716	27 713

Supplementary Figure 14: **Centromere calls for *Z. rouxii***

Smoothed *trans* contact counts (with $\sigma = 40$ kb) overlaid with Centurion's centromere calls (black line). White lines represent chromosome boundaries.

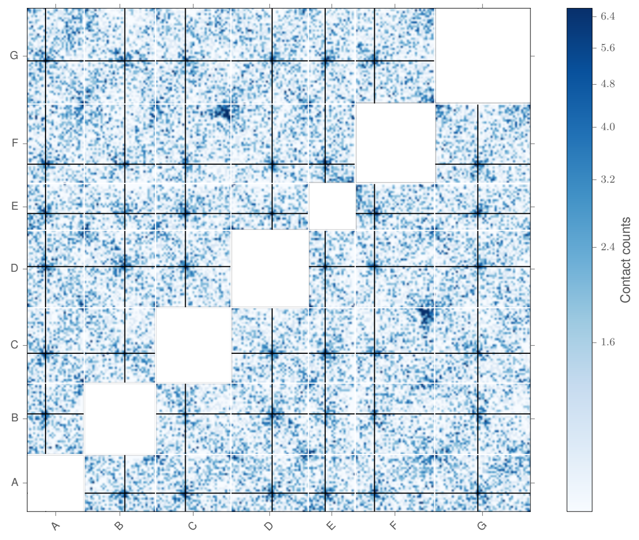

Supplementary Table 15: ***Z. rouxii* centromere calls, ground truth and errors**

Chromosome	Ground truth	20 kb		40 kb	
		Call	Error	Call	Error
A	369 077 - 369 243	353 671	15 406	360 198	8879
B	788 730 - 788 896	782 871	5859	796 526	7630
C	581 961 - 581 795	582 298	503	586 407	4612
D	807 719 - 807 885	804 544	3175	808 613	728
E	335 012 - 334 844	333 919	1093	330 354	4658
F	372 701 - 372 867	376 542	3675	378 835	5968
G	852 551 - 852 385	841 169	11 382	847 614	4937

Supplementary Figure 15: **Centromere calls for *P. pastoris***

Smoothed *trans* contact counts (with $\sigma = 40$ kb) overlaid with Centurion's centromere calls (black line). White lines represent chromosome boundaries.

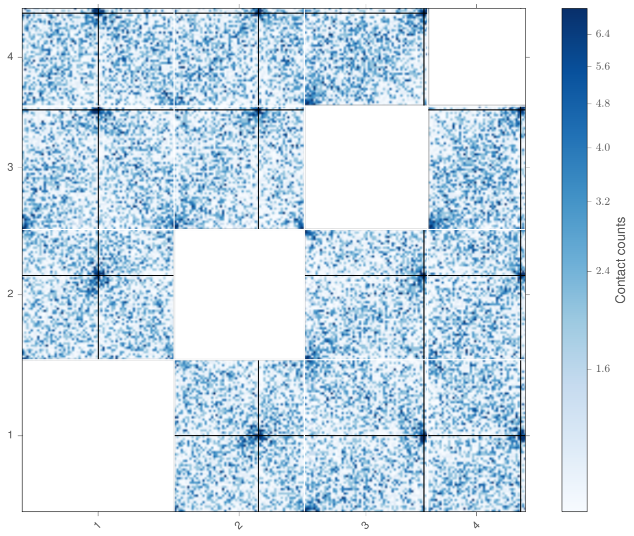

Supplementary Table 16: ***P. pastoris* de novo centromere calls**

Chromosome	20 kb call	40 kb call
1	1 408 908	1 404 605
2	1 556 231	1 556 450
3	2 226 823	2 209 846
4	1 719 280	1 712 207

Supplementary Figure 16: **Centromere calls for *E. gossypii***

Smoothed *trans* contact counts (with $\sigma = 40$ kb) overlaid with Centurion's centromere calls (black line). White lines represent chromosome boundaries.

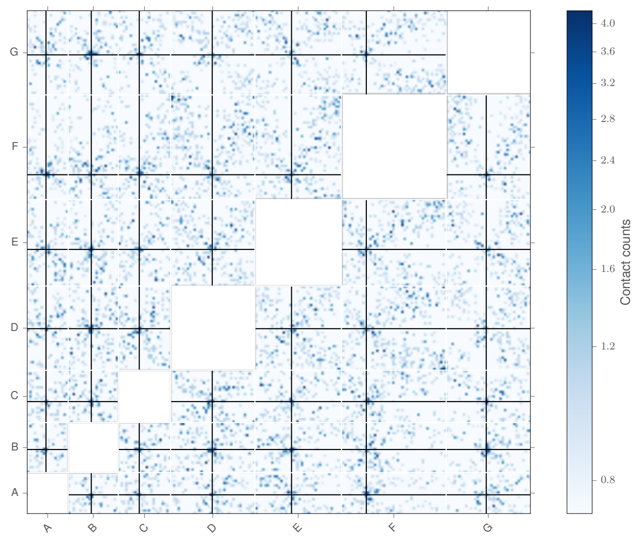

Supplementary Table 17: ***E. gossypii* de novo centromere calls**

Chromosome	20 kb call	40 kb call
A	338 620	329 920
B	399 593	406 065
C	357 805	368 603
D	717 357	730 541
E	601 683	643 434
F	491 379	436 967
G	718 695	704 238

Supplementary Figure 17: **Centromere calls for *K. wickerhamii***

Smoothed *trans* contact counts (with $\sigma = 40$ kb) overlaid with Centurion's centromere calls (black line). White lines represent chromosome boundaries.

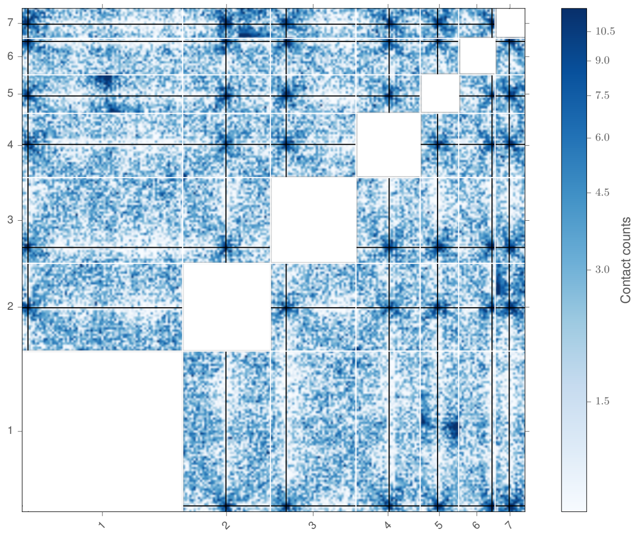

Supplementary Table 18: ***K. wickerhamii* de novo centromere calls**

Chromosome	20 kb call	40 kb call
1	107 436	108 558
2	807 861	809 232
3	290 904	295 270
4	618 467	620 001
5	323 875	325 408
6	622 741	623 503
7	266 146	264 963

Supplementary Figure 18: **Centromere calls for *L. waltii***

Smoothed *trans* contact counts (with $\sigma = 40$ kb) overlaid with Centurion's centromere calls (black line). White lines represent chromosome boundaries.

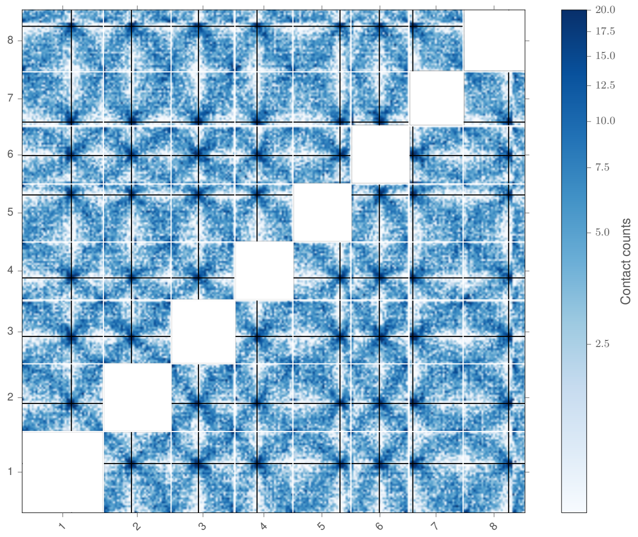

Supplementary Table 19: ***L. waltii* de novo centromere calls**

Chromosome	20 kb call	40 kb call
1	1 028 089	1 017 632
2	587 954	580 429
3	565 659	566 391
4	454 852	457 549
5	971 551	973 444
6	589 260	587 604
7	80 178	79 371
8	935 869	941 058

Supplementary Figure 19: Centromere calls for *S. paradoxus*

Smoothed *trans* contact counts (with $\sigma = 40$ kb) overlaid with Centurion's centromere calls (black line). White lines represent chromosome boundaries.

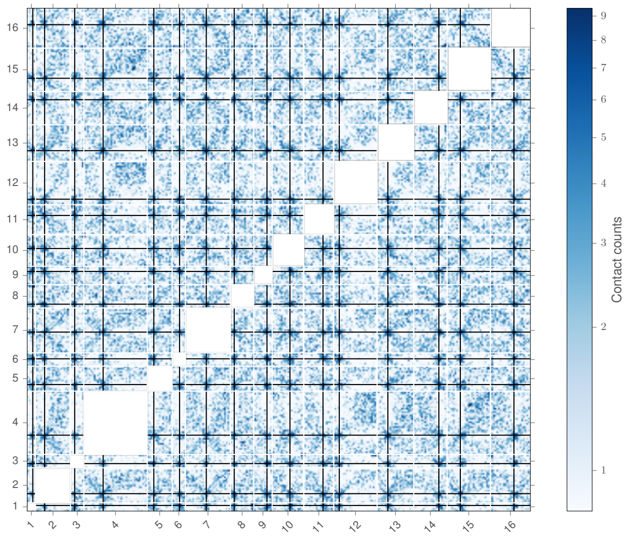

Supplementary Table 20: *S. paradoxus* de novo centromere calls

Chromosome	20 kb call	40 kb call
1	138 267	134 145
2	222 275	217 965
3	100 865	100 219
4	458 330	462 705
5	154 493	159 191
6	178 545	178 292
7	494 545	499 430
8	87 986	89 589
9	317 389	316 309
10	414 694	427 637
11	455 761	461 330
12	128 761	131 623
13	256 588	258 273
14	601 000	602 387
15	316 026	317 082
16	564 137	564 849

Supplementary Figure 20: **Centromere calls for *S. stipitis***

Smoothed *trans* contact counts (with $\sigma = 40$ kb) overlaid with Centurion's centromere calls (black line). White lines represent chromosome boundaries.

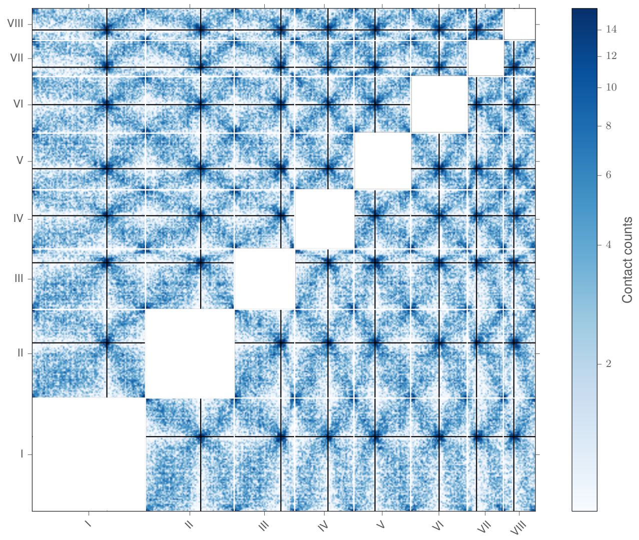

Supplementary Table 21: ***S. stipitis* de novo centromere calls**

Chromosome	20 kb call	40 kb call
I	2 309 980	2 320 851
II	1 708 033	1 717 536
III	1 451 523	1 448 891
IV	1 039 527	1 032 779
V	655 745	654 571
VI	893 268	886 606
VII	279 875	289 537
VIII	325 140	330 100

Supplementary Figure 21: **Replication timing profile across the *P. pastoris* genome**

Adapted from Figure 4 and Supplementary Figure 6 in Liachko et al. [2014]. The curve represents the smoothed copy number ratio of genomic DNA in cells undergoing S phase versus cells in G1 phase. Peaks correspond to positions of early replication (replication origins) and valleys represent late replicating regions (replication termini). Circles represent potential replication initiation sites. The positions of centromeres predicted by Centurion are indicated as red lines.

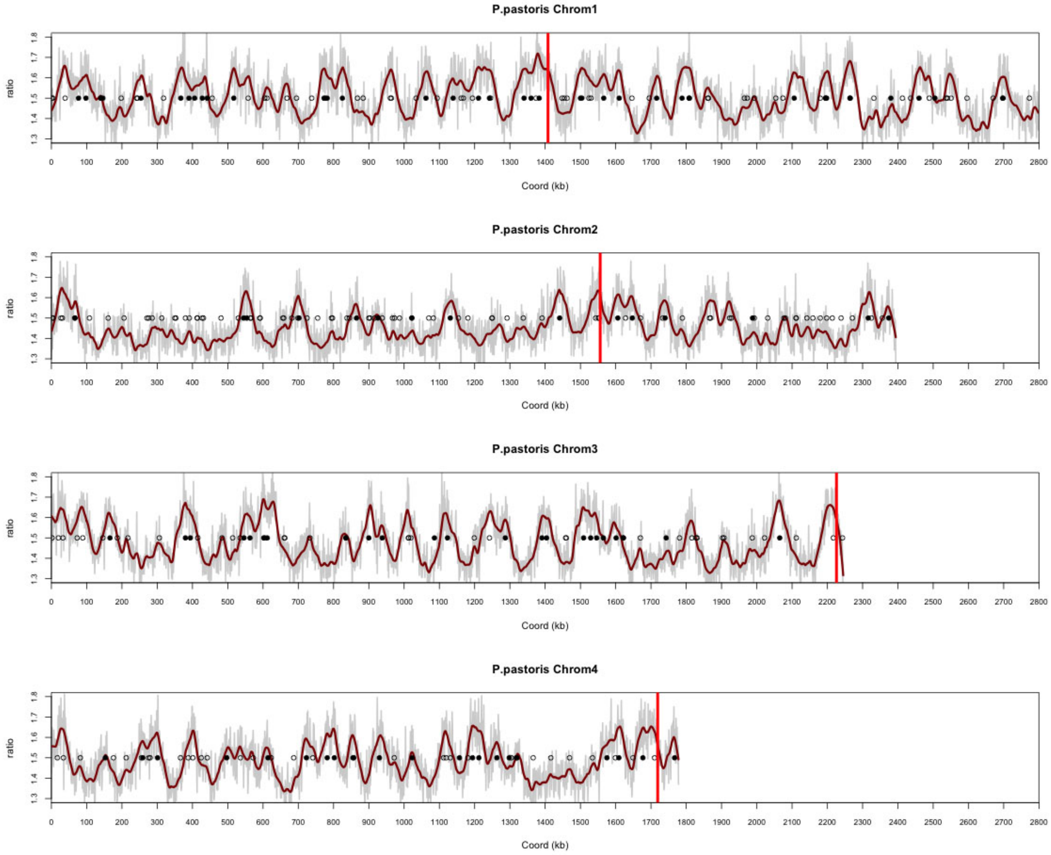

Supplementary Notes

Initializing the optimization

The optimization problem being non convex, the local minimum found by the algorithms depends on the starting point. We therefore implemented heuristics to initialize the optimization with several sets of centromere positions. Our implementation of Centurion also allows the user to specify the starting point (*ie* the rough centromere location).

For each chromosome, the centromeric regions are expected to be enriched in *trans* contact counts. We thus seek a few local maxima in the marginalized *trans* contact count profile $p(i) = \sum_{i,j|\mathcal{B}(i)\neq\mathcal{B}(j)} c_{ij}$ for each chromosome. In order to select only k candidates per chromosome, we smooth the contact counts profile p with a Gaussian filter of parameter σ, setting σ such that there are k peaks in the profile. We consequently obtain a set of k centromere candidates per chromosome, and thus can initialize the optimization with all possible combination of these candidates.

To reduce computation time, we implemented a set of heuristics to decrease the number of candi-

dates. First, note that the higher the contact count enrichment peak is, the more likely a candidate is to be the in the centromeric region. Second, remember that we attempt to jointly optimize centromeres location: we optimize L variables at once, L being the number of chromosomes, and each variable corresponding to a chromosome position. To reduce the number of candidates per chromosome, we first compute a baseline, by performing the optimization using as starting point the set of most likely candidate for each of our L chromosomes (the candidate with the highest peak for each of the chromosomes). Then, for each candidate p_i of the l-th chromosome, we perform the optimization once, using as starting point the set of most likely candidates, replacing the l-th one by p_i. If the objective function value is higher than our baseline (thus, using p_i as a candidate for chromosome l did not improved the fit), we remove the candidate from our list. We thus reduced the number of candidates in a small number of steps and can proceed with initializing the optimization with the all possible combination of this reduced set of candidates. Our implementation allows the user to specify whether or not to perform this filtering step.

References

I. Liachko, R. A. Youngblood, K. Tsui, K. L. Bubb, C. Queitsch, M. K. Raghuraman, C. Nislow, B. J. Brewer, and M. J. Dunham. GC-rich DNA elements enable replication origin activity in the methylotrophic yeast *Pichia pastoris*. *PLoS Genet.*, 10(3):e1004169, Mar 2014.

H. Marie-Nelly, M. Marbouty, A. Cournac, G. Liti, G. Fischer, C. Zimmer, and R. Koszul. Filling annotation gaps in yeast genomes using genome-wide contact maps. *Bioinformatics*, pages 2105–2113, 2014.
